# The Physicochemical Properties and Structure of Mung Bean Starch Fermented by *Lactobacillus plantarum*

**DOI:** 10.3390/foods13213409

**Published:** 2024-10-26

**Authors:** Zhen Huang, Yisi Li, Tian Guo, Li Xu, Jieyao Yuan, Zuyin Li, Cuiping Yi

**Affiliations:** 1 College of Social Development and Management, Hunan Women’s University, Changsha 410004, China; 2 School of Food Science and Bioengineering, Changsha University of Science and Technology, Changsha 410114, China

**Keywords:** fermentation, *Lactobacillus plantarum*, mung bean starch, physicochemical properties, structure

## Abstract

Understanding the relationship between gel formation and the hierarchical structure of mung bean starch fermented by *Lactobacillus plantarum* has potential value for its green modification and quality improvement. The variations in characteristics, including gelation characteristics, starch chain, and the molecular order degree of mung bean starch fermented by different *L. plantarum*, were compared. The results show that in the gelation process, starch began to disintegrate at 65 °C, indicating a critical temperature for structural changes. Compared with the control group, although the effects of different *L. plantarum sources* on mung bean starch varied, notable improvements were observed in water absorption across all groups of fermented starch, along with reduced free water-soluble substances and enhanced anti-expansion ability. This led to the easier formation of gels with higher viscosity, primarily attributed to decreased crystallinity, increased short-chain amylopectin tendency, an elevated amylose content, and enhanced short-range order when microorganisms acted on the crystallization zone. In conclusion, although *L. plantarum* came from different sources, its action mode on mung bean starch was similar, which could enhance the gel structure.

## 1. Introduction

Mung bean, a significant leguminous plant, boasts starch as one of its primary components, constituting approximately 50–60% of its total components [1]. In the granules of mung bean starch, amorphous and semi-crystalline growth rings composed of amylose and amylopectin are alternately filled. The content of amylose and amylopectin and the crystal structure in starch will affect the setback viscosity, peak viscosity, and final viscosity of starch, thus affecting the quality of starch-based foods [2]. Although natural mung bean starch has been widely applied as an additive in starch-based foods, such as noodles and rice noodles [3,4], due to its high amylose content and excellent gel-forming ability, there are still problems such as low heat resistance, high dehydration rate, and low solubility, limiting the further development of mung bean starch in the starch-based food field [5].

One useful approach for improving the quality of foods based on starch is fermentation. It modifies the structure of starch through the metabolic action of microorganisms and the production of extracellular enzymes, thus improving the quality properties of the product. Lactic acid bacteria represent one of the most extensively utilized microbial agents in traditional food fermentation processes involving cereals and legumes [6]. For instance, during the fermentation of mung bean starch by lactic acid bacteria, the percentage of long amylopectin B and B1 chains increased, and the viscosity and gelatinization enthalpy decreased [7]. In addition, compared with natural fermentation, the purebred starter has the advantages of stability, controllability, and safety [8], among which *Lactobacillus plantarum* in lactic acid bacteria is considered to have strong potential in improving the starch structure and other uses [9]. The changes in the properties of glutinous proso millet starch before and after fermentation by *L. plantarum* were compared by Bian et al. [10], and it was found that pure fermentation could improve the starch properties more effectively, such as by increasing crystallinity and the swelling degree and causing amylopectin to tend towards short-chainization. Additionally, the effects of *L. plantarum* on the quality of starch-based products include enhancing the gel strength, inhibiting product retrogradation, and prolonging the shelf life [11,12]. However, there is a paucity of research investigating the effects of *L. plantarum* derived from various sources on the structural and physicochemical properties of mung bean starch. A comparative analysis of the applicability and influence rule of starter cultures is helpful to the processing and utilization of mung beans.

This study investigated the effects of *L. plantarum* isolated from three kinds of traditional Chinese foods on fermented mung bean starch, encompassing the analysis of gel water absorption and the forming properties, physicochemical properties, and ordered structure. The findings from this study are expected to contribute to a better understanding of the mechanism by which the starch structure of mung beans changes during fermentation as well as to give a theoretical foundation for the production of high-value mung beans.

## 2. Material and Methods

### 2.1. Materials

Mung beans were provided by Yunzhou Farmers’ Cooperative (Datong, Shanxi, China). Three types of *L. plantarum* used in the fermentation of mung beans, including *L. plantarum* 23169 isolated from sour dough and *L. plantarum* 22699 isolated from sauerkraut, were purchased from the China Center for Type Culture Collection (CCTCC); *L. plantarum* YI-Y2013 (CCTCC NO: M2017533) was isolated from the natural fermentation liquid of *indica* rice at the Jinjian rice noodle factory (Changde, Hunan, China) by our research team. MRS broth culture medium was purchased from Guangdong Huankai Microbiological Technology Co., Ltd. (Guangzhou, Guangdong, China). Sodium hydroxide, dimethyl sulfoxide, hydrochloric acid, and other reagents used for starch extraction and composition determination were analytically pure and purchased from Sinopharm Group Chemical Reagents Co., Ltd. (Shanghai, China).

### 2.2. Preparation of Mung Bean Flour 

The fermentation of mung beans was consistent with the method described in our previous study [9]. In short, the mixture ratio of mung beans and sterile water was 1:3 (g/g), and the *L. plantarum* was inoculated at a concentration of 10^8^ CFU/mL based on the volume of sterile water, fermented at 37 °C for 48 h, and then washed, freeze-dried, and crushed to obtain fermented mung bean flour. In the unfermented group, mung beans were steeped in a solution containing 0.17 g/L clindamone and 0.2 g/L chloramphenicol in the same proportion at 4 °C, and the other processes were the same. The total starch contents of unfermented and three kinds of fermented mung bean flour were 54.25% (Control), 54.86% (*L. plantarum* YI-Y2013), 57.48% (*L. plantarum* 22699), and 56.14% (*L. plantarum* 23169), respectively. 

### 2.3. Extraction of Starch from Mung Bean Flour

Minor adjustments were made to an existing method [13] for extracting the starch from mung bean flour. After dispersing the mung bean flour in a 0.2% NaOH solution (1:4, *w*/*v*), it was kept at 25 °C for 12 h, the supernatant was removed by centrifugation, and the precipitation was repeatedly washed to a neutral state by water. The starch was dried for 24 h at 40 °C in an oven, ground, and passed through an 80-mesh sieve. The purity values of unfermented and three kinds of fermented mung bean starch were 89.93% (Control), 86.91% (*L. plantarum* YI-Y2013), 84.57% (*L. plantarum* 22699), and 83.37% (*L. plantarum* 23169), respectively.

### 2.4. Hydration Properties

With a minor modification, the method proposed in [14] was used to determine the hydration properties of the mung bean starch. One gram (*M*_0_) of mung bean starch was added into 20 mL distilled water, stirred well, and heated for 10 min in a water bath at 90 °C. It was centrifuged at 3580× *g* for 10 min after cooling. The precipitate mass after centrifugation was *M*_3_. The supernatant was transferred to an aluminum box with a constant weight (*M*_1_) and dried at 105 °C to constant weight, and the weighing mass was *M*_2_. The following formulas were used to calculate the swelling power (SP), water solubility index (WSI), and water absorption index (WAI):

SP = *M*_3_/[*M*_0_ × (1 − WSI)] (1)

WSI = (*M*_2_ − *M*_1_)/*M*_0_ × 100% (2)

WAI = *M*_3_/*M*_0_ (3)

### 2.5. Pasting Properties

The pasting properties of mung bean starch was determined by a rapid viscosity analyzer (RVA 4500; Perten, Hägersten, Sweden). About 3 g of sample (corrected by 12% moisture content) was added to 25 mL deionized water to make a suspension for determination. The specific parameters were as follows: heating to 50 °C for 1 min; heating to 95 °C for 2.5 min; and finally cooling down to 50 °C for 2 min with a 12 °C/min heating and cooling rate [15].

### 2.6. X-Ray Diffraction (XRD)

The XRD (D8 Advance Brook, Brook, Karlsruhe, Germany) pattern of mung bean starch was determined. At a speed of 10°/min, the diffraction angle 2θ ranged from 5° to 40°. The JADE software 5.0 (Materials Date Inc., Livermore, CA, USA) was utilized to determine the relative crystallinity of the mung bean starch by dividing the sample peak area by the overall diffractogram area.

### 2.7. Fourier Transform Infrared Spectra (FT-IR)

After precisely weighing the mung bean starch, it was combined with KBr at a 1:100 ratio. The mixture was further finely ground in a mortar and pressed into clear slices with a tablet press. The FT-IR spectra was scanned and obtained between 4000 and 400 cm^−1^, and the spectrum was deconvolved using Ominic 8.5 (half-width of 18 cm^−1^; resolution enhancement of 1.9). The peak area ratio of R1047/1022 cm^−1^ was calculated to determine the molecular order (DO), and R995/1022 cm^−1^ was calculated to indicate the double helicity (DD).

### 2.8. Amylose Determination 

According to the AACC method 61-03.01 [16], iodine colorimetry was used to determine the apparent amylose concentration in mung bean starch. The standard curve was conducted with potato amylose as the standard. The content of amylose in mung bean starch was calculated by measuring the absorbance of starch–iodine complex solution at 720 nm.

### 2.9. Distribution of Amylopectin Chain Length

According to a previous study [14], the chain length distribution of mung bean amylopectin was ascertained. The standard curve was determined using pullulan standards of different relative molecular weights. Depending on the amount of glucosides, the amylopectin chain length distribution was separated into four categories: FA (DP 6–12), Fb_1_ (DP 13–24), Fb_2_ (DP 25–36), and Fb_3_ (DP 37–60). The specific sample processing steps were as follows: after 50 mg of dry mung bean starch was dissolved in 10 mL of 90% (*v*/*v*) dimethyl sulfoxide, the mixture was magnetically agitated for 1 h in a boiling water bath and then for 24 h at room temperature. An amount of 6 mL of anhydrous ethanol and 1 mL of the previously mentioned solution were mixed and centrifuged for 20 min at 3580× *g*, and the supernatant was disposed of, and 9 mL of boiling ultrapure water was added. After boiling in a boiling water bath for 10 min, the centrifuge tube was transferred to 50 °C water, and then 1 mL of glycine–hydrochloric acid buffer (pH 3.5) was added. After mixing evenly, 2 μL of isoamylase (200 U/mL) was added, and the water bath was continued for 48 h. Finally, it was transferred to boiling water and boiled for 15 min. Before injection into the high-performance size-exclusion chromatography (HPSEC) system (Waters 2695, Waters Corporation, Milford, CT, USA), it was filtered through a water-washed filter (0.22 μm) and injected while hot. During the determination process, the experiment was conducted with ultrapure water as the mobile phase, a 50 °C column temperature, a 30 °C detector temperature, a 1.0 mL/min flow rate, and a 20 μL injection volume.

### 2.10. Statistical Analysis

Every measurement was made in at least three duplicates. Using SPSS 26.0 (SPSS Inc., Chicago, IL, USA), statistical analyses were performed using the least significant difference (LSD) test, with *p* < 0.05 indicating significance, and a one-way analysis of variance (ANOVA) was conducted. 

## 3. Results and Discussion

### 3.1. Gel Properties of Natural and Fermented Mung Bean Starch

#### 3.1.1. Hydration Properties

**Figure 1:**
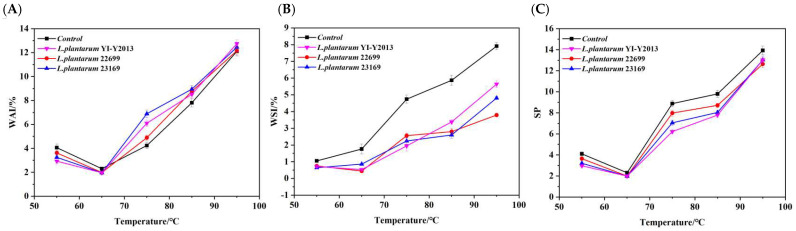
The hydration capacity of natural and fermented mung bean starch. (**A**) The water absorption index (WAI); (**B**) the water solubility index (WSI); and (**C**) the swelling power (SP). Note: Each value in the figure is a mean of three replicate determinations.

The hydration parameters of mung bean starch at various temperature points during the 55–95 °C heating process were measured to comprehend the dynamic change in water absorption and expansion during the production of mung bean starch gel. The changes in hydration characteristics of mung bean starch both before and after *L. plantarum* fermentation are shown in Figure 1. The data in the figure are presented as shown in Table S1. The intricate interactions between starch chains and water during the heat gelatinization process are reflected in the WAI, WSI, and SP of starch. According to [17], the hydration properties of starch were directly associated with its molecular weight/size, chain length, amylose content, relative crystallinity, and other structural features.

The WAI serves as an indicator of the water-holding capacity of various substances. All samples exhibited a trend characterized by an initial significant decrease in the WAI, followed by an increase with the rising temperature (*p* < 0.05). At 65 °C, the samples displayed the lowest water absorption capacity, potentially indicating the critical point at which the starch structure was compromised. During the heating process, the crystal structure of starch is disrupted due to the breakage of hydrogen bonds [18]. Notably, around 75 °C, variations in the water absorption capacity at the gelatinization temperature were observed among the different samples, likely attributed to fermentation-induced alterations in the amylopectin/amylose ratio of the starch matrix. The differences in the SP of fermented mung bean starch may be due to the varying degrees of starch modification by different sources of *L. plantarum*. The WSI of the fermented and unfermented samples showed a significant increasing trend with the temperature (*p* < 0.05), and the control group had the highest WSI, followed by *L. plantarum* YI-Y2013, *L. plantarum* 23169, and *L. plantarum* 22699. This might be due to the fact that during fermentation, starch was hydrolyzed into small molecular sugars by the acid and enzyme action of *L. plantarum*. These monosaccharides and disaccharides are subsequently utilized by microorganisms to provide energy production, thereby diminishing the concentration of soluble substances [10]. According to prior research [19], fermentation changed the structure and function of starch, exposing more hydrophobic groups, which resulted in a decrease in the water solubility of fermented starch. A greater SP typically indicated granules with lower levels of intermolecular associative interactions [20]. Compared with the control group, the SP of three *L. plantarum* fermented samples decreased slightly, which was attributed to the internal rearrangement of starch particles caused by fermentation, leading to the leaching of amylose. Amylose prevented the starch particles from swelling, which was in line with previous research [21], which resulted in stronger anti-swelling and structural strength [22]. 

#### 3.1.2. Pasting Properties

The gelatinization of starch largely influences the quality of starch food and has a profound impact on starch applications. The pasting properties of both fermented and unfermented mung bean starch are presented in Table 1. The trough viscosity (TV), peak viscosity (PV), and final viscosity (FV) of fermented mung bean starch exhibited significant increases (*p* < 0.05). Viscosity serves as an indirect indicator reflecting the water absorption of starch. Amylopectin with a high branching degree is more conducive to the absorption and retention of water. The observed increase in viscosity after fermentation might be due to the acid and enzyme acting on the amorphous region of starch during fermentation; amylopectin with long chains was broken down into amylose and amylopectin with short chains. This was in line with the findings obtained for wheat starch [23]. Zhang et al. [24] found that as the degree of polymerization of branched starch decreased and the molecular content of short chain starch increased, the samples had higher PV, TV, and FV values alongside a lower gelatinization temperature. Conversely, opposing results were observed in yam starch [25] and potato starch [26]. The decrease in viscosity correlated with the accumulation of short chains in starch depending on the degree of damage to the internal structure of starch caused by fermentation and the fine structure of starch. The setback (SB) value represents the rearrangement of amylose recovered from enlarged starch granules after chilling [27]. After fermentation by *L. plantarum*, the SB value of mung bean starch increased, possibly due to the increase in the amylose content. Moreover, the rearrangement of short chains promoted the formation of a double-helix structure, which then led to the increase in the SB. The higher the breakdown (BD) value, the easier it is for starch to break when gelatinized. After fermentation, the BD values increased across various samples, but only *L. plantarum* YI-Y2013 was significant (*p* < 0.05).

There was not a noticeable variation in the gelatinization time of fermented mung bean starch, but a slight increase in the gelatinization temperature was observed. This may be due to some low-molecular-weight substances, such as monosaccharide, competing with starch for available water [28]. It was also possible that the organic acids generated during the fermentation process disrupted the complete structure of the crystallization zone, and the slow degradation of the acid on the crystallization zone led to an increase in the gelatinization temperature of fermented mung bean starch [29]. In general, *L. plantarum* fermentation made mung bean starch more easily absorb water, gelatinize at a higher temperature, and reconstitute into a denser gel with higher viscosity after cooling.

### 3.2. Ordered Structure of Natural and Fermented Mung Bean Starch

#### 3.2.1. XRD Analysis

**Figure 2:**
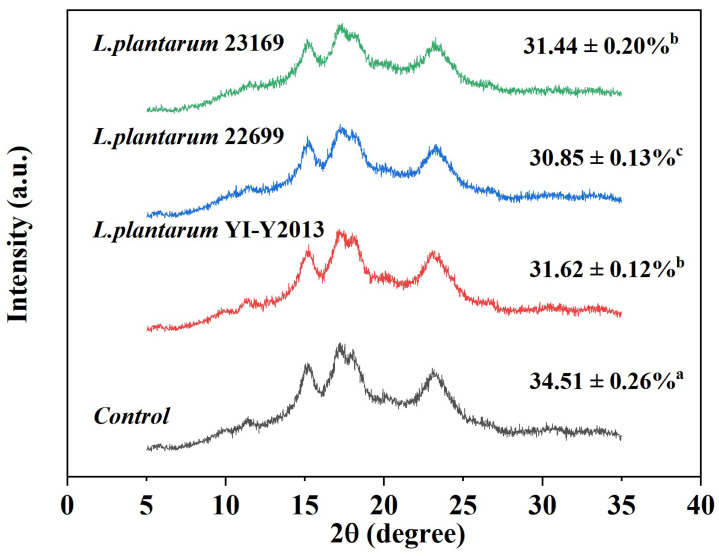
The XRD patterns of natural and fermented mung bean starch. Note: Each value in the figure is a mean of three replicate determinations. Different lowercase letters in the figure signify significant differences (*p* < 0.05).

The long-range organized crystal structure of starch can be assessed with XRD [30]. The XRD patterns of mung bean starch fermented by three strains of *L. plantarum* are shown in Figure 2. All mung bean starch samples exhibited five strong diffraction peaks at 5.6°, 15.2°, 17.2°, 18.2°, and 23.2°, which was a typical C-type starch crystallization mode [31]. The results demonstrate that the crystal-type structure of the starch remained unchanged throughout the fermentation process, and similar outcomes were observed for rice starch [32] and glutinous rice starch [33]. The unfermented starch samples displayed a relative crystallinity of 34.51%, while the fermented starch samples had relative crystallinities of 31.62%, 30.85%, and 31.44%, respectively. Compared to the unfermented samples, the fermented mung bean starch exhibited a considerably reduced relative crystallinity. The findings suggest that *L. plantarum* initially hydrolyzed the loose branched starch outside the crystalline region through acid or enzyme hydrolysis. As the fermentation time increased, the structure of the crystalline zone was damaged, resulting in a decrease in the crystalline area. Wang et al. [34] also reported that fermentation disrupted the crystalline structure of starch, resulting in a decrease in relative crystallinity. The fermentation samples of *L. plantarum* from various sources showed no discernible differences, indicating that the three types of *L. plantarum* exerted comparable effects on the long-range ordered structure of mung bean starch.

#### 3.2.2. FT-IR Analysis

**Figure 3:**
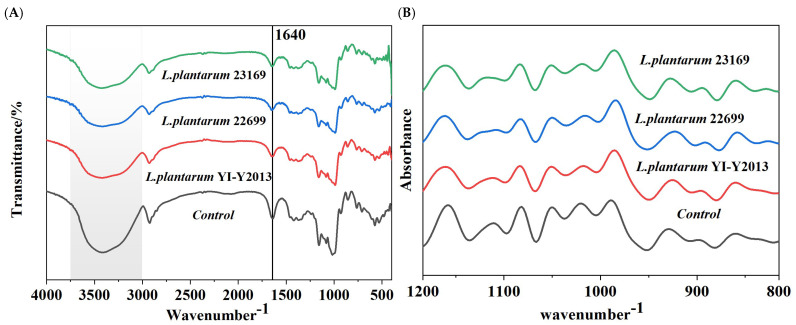
The FT-IR spectra (**A**) and deconvoluted FT-IR spectra (**B**) of natural and fermented mung bean starch. Note: The gray portion in (**A**) constitutes the hydrogen bond stretching vibration region ranging.

The spectral transformation and short-range molecular order of fermented mung bean starch in the 400–4000 cm^−1^ range were investigated using FT-IR. Figure 3A displays the infrared spectra of starch both prior to and following fermentation. It can be observed that both fermented mung bean starch and the control sample had similar patterns without generating new diffraction peaks, indicating that no new functional group was formed during fermentation. The deconvoluted FT-IR spectra of the samples in the 800–1200 cm^−1^ range are shown in Figure 3B. To determine the short-range order of starch, the ratio of the ordered crystalline area to the amorphous region close to the starch granule (R1047/1022) was typically utilized to measure the DO of the vicinity, and R995/1022 was used to quantify the DD, both of which were counted in Table 2.

The characteristic peak of the fermentation sample at the 3000–3600 cm^−1^ range suggests the tensile vibration of O-H [35], signifying that *L. plantarum* fermentation weakened the hydrogen bonding between intra-starch molecules and water molecules within the spiral region of the starch crystal. The moisture content of the samples and the vibrational dynamics of the adsorbed water molecules in the non-crystalline region were associated with the band at 1640 cm^−1^ [36]. The control group had the highest peak intensity at this wavelength, which might denote that the amorphous region of fermented mung bean starch was destroyed by acids or enzymes, and fermentation prevented the binding of water molecules in the non-crystalline zone, weakening the peak strength of fermented mung bean starch at 1640 cm^−1^. Compared with natural starch, the DO values of fermented mung bean starch all increased. It might be implied that the starch amorphous region was first hydrolyzed by enzymes secreted by microorganisms, which increased the scale of the ordered structure on the surface of starch particles. Additionally, a more flexible section of amylopectin was produced by the breakdown of the amorphous region of starch molecules. The same results could be observed in the co-fermentation of rice starch with yeast and lactic acid bacteria [32]. Additionally, it was observed that the DD value of fermented mung bean starch increased because fermentation hydrolyzed the longer amylopectin into short chains, which were responsible for the formation of a double helix [37]. Naturally fermented highland barley starch produced the same outcome [38].

### 3.3. Fine Chain Structures of Natural and Fermented Mung Bean Starch

#### 3.3.1. Amylose Content

The distribution of amylose and amylopectin constitutes a critical aspect of the fine structure of starch, as illustrated in Table 3. The content of amylose in natural starch was closely associated with its physicochemical properties [39]. The results indicate that the amylose content of fermented mung bean starch exhibited an increasing trend compared to the control group. This behavior might be explained by *L. plantarum* producing organic acids or enzymes during the fermentation process. These compounds might lead to the cleavage of short chains of amylopectin in the amorphous region, resulting in the formation of new small-molecule amylose and consequently augmenting the amylose content [40]. A similar increase in the amylose content was observed in fermented yam starch [25]. 

In addition, the decrease in crystallinity that was observed (Figure 2) also reflected that the dense crystalline region of the starch in mung bean was degraded by *L. plantarum*, which was mainly composed of the spiral structure of amylopectin, indicating that fermentation disrupted the double-helix structure of the crystalline area, leading to the degradation of long-chain amylopectin and consequently resulting in the formation of new amylose.

#### 3.3.2. Amylopectin Chain Length Distribution

The amylopectin chain length distribution was utilized to study the impact of fermentation on the fine structure alterations of amylopectin [10]. Table 3 presents the distributions of amylopectin chain lengths for both natural and fermented mung bean starch. The chain length of amylopectin is divided into FA, Fb_1_, Fb_2_, and Fb_3_ according to the amount of glucose glycogen. Fb_2_ and Fb_3_ represent long B chains, and FA and Fb_1_ represent short chains [41], while chains Fb_2_ and Fb_3_ mostly participate in the amorphous zone, and chains FA and Fb_1_ are present in the crystalline region. The content of long-chain Fb_3_ reduced, while the content of FA increased according to the data, and there was no significant difference (*p* < 0.05) between the contents of chains Fb_1_ and Fb_2_ before and after fermentation, which is consistent with the above assumption. The FA and Fb_1_ of fermented yam starch [25] demonstrated a tendency to first drop and then increase, indicating a strong correlation between the kind of starch, fermentation duration, and the variation in chain length distribution. The long chain of fermented starch is broken down, and short chains accumulate as a result of the branching long chains in the amorphous area being hydrolyzed by acid and enzymes. A similar variation in the branch chain length was also observed in fermented sweet potato starch [42]. Another reason for the reduction in long B chains may be that enzymes degraded starch into oligosaccharides, which could serve as an energy source for microbial growth. Among all the fermented samples, the *L. plantarum* 23169 fermented mung bean starch had the highest degradation. This might indicate that *L. plantarum* 23169 had the strongest ability to utilize starch. Diverse genetic backgrounds and phenotypic variations were observed among *L. plantarum* sourced from different origins [43]. These disparities consequently exerted an influence on their enzymatic activities and acidogenic capabilities during the fermentation process.

According to the findings of Chen et al. [44] the amount of long amylopectin was invertedly associated with the temperature at which starch gelatinized, but positively correlated with the content of amylose. Therefore, the change in the distribution patterns of amylose and amylopectin in Table 3 might be related to the increase in the starch gelatinization temperature of fermented mung bean in Table 1. This was also confirmed by the low expansion power of the aforementioned fermented starch and also indicates enhanced boiling resistance. 

### 3.4. A Discussion of the Structural Alterations that Occur in Mung Bean Starch During Fermentation

**Figure 4:**
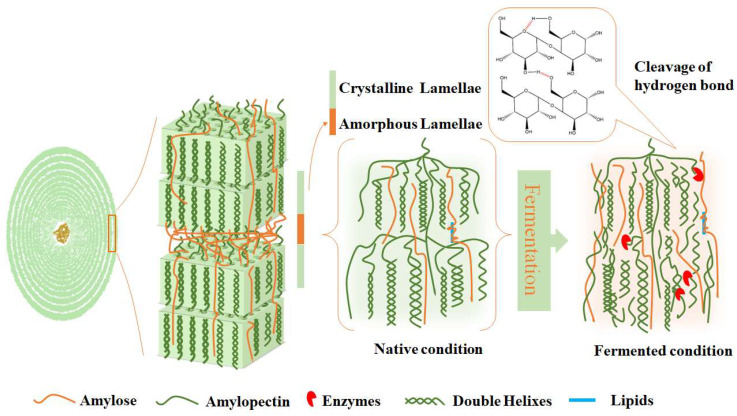
A schematic presentation of the changes in the mung bean starch structure during *L. plantarum* fermentation.

The structure change mechanism of mung bean starch fermented by *L. plantarum* is shown in Figure 4. The mung bean starch granules underwent 48 h of fermentation before a hydrogen bonding network was broken by the acid and enzyme generated by *L. plantarum*, thus destroying the crystal structure of the starch, and there was a significant decreasing trend in the crystallinity of the fermented starch (Figure 2). The long chains in the amorphous region of mung bean starch were hydrolyzed into short chains, and the newly generated short chains were recombined to form a new double-helix structure, increasing the order degree, double-helix structure (Table 2 and Figure 3), and setback value of fermented mung bean starch (Table 1). The destruction of the amorphous zone led to large leaching of amylose (Table 3), which impeded the entry of water and inhibited the swelling of starch granules, and all the values of pasting viscosity of starch increased with the increase in the amylose content. *L. plantarum* fermentation could disrupt the crystalline structure of mung bean starch, significantly improve both the short-chain amylopectin and amylose contents, inhibit starch solubilization, and promote an increase in double-helix formation along with the short-range ordered structure.

## 4. Conclusions

The effects of *L. plantarum* fermentation from three different sources on the gel formation of mung bean starch were studied with a focus on the microstructural changes related to the starch order and fine structure. After the fermentation of mung bean starch by *L. plantarum*, water absorption increased, while swelling and water solubility decreased; concurrently, the gel viscosity, breakdown, and setback values all increased, indicating that forming gel and a compact structure was easier. The microscopic mechanism revealed that, as the main component of the crystal region of fermented mung bean starch, the short chain of amylopectin was formed through the hydrolysis of the lengthy chain. The relative crystallinity decreased and changed to an amorphous region, the content of amylose increased, and the degree of order increased in the short range. This study has some relevance for the development, application, and regulation of foods based on mung bean starch and offers a theoretical foundation for mung bean fermentation.

## Figures and Tables

**Table 1:** The pasting properties of natural and fermented mung bean starch.

Samples	PV/cP	TV/cP	FV/cP	BD/cP	SB/cP	PT/min	GT/°C
Control	5088 ± 0.71 ^c^	1607 ± 9.19 ^c^	4428 ± 164.05 ^c^	2345 ± 4.95 ^b^	1703 ± 10.61 ^c^	4.57 ± 0.05 ^a^	75.20 ± 0.07 ^b^
*L. Plantarum* YI-Y2013	5364 ± 17.68 ^b^	3019 ± 12.73 ^b^	4722 ± 2.12 ^b^	3481 ± 9.90 ^a^	2822 ± 173.24 ^a^	4.60 ± 0.00 ^a^	78.75 ± 0.49 ^a^
*L. Plantarum* 22699	5818 ± 47.38 ^a^	3433 ± 65.76 ^a^	5176 ± 61.52 ^a^	2367 ± 43.84 ^b^	1743 ± 4.24 ^c^	4.50 ± 0.04 ^a^	76.40 ± 0.49 ^b^
*L. Plantarum* 23169	5464 ± 72.12 ^b^	2966 ± 185.97 ^b^	5069 ± 26.87 ^a^	2499 ± 258.09 ^b^	2104 ± 159.12 ^b^	4.57 ± 0.05 ^a^	77.98 ± 0.53 ^a^

All data are shown as the mean ± SD, and each value is a mean of three replicate determinations. Different letters in the same column indicate significant differences in the data (*p* < 0.05). PV, peak viscosity; TV, trough viscosity; FV, final viscosity; BD, breakdown; SB, setback; PT, pasting time; GT, gelatinization temperature.

**Table 2:** The short-ordered structures of natural and fermented mung bean starch.

Sample	Short-Ordered Parameters	
	DO (R1047/1022)	DD (R995/1022)
Control	0.848 ± 0.056 ^b^	1.150 ± 0.080 ^c^
*L. Plantarum* YI-Y2013	0.981 ± 0.020 ^a^	1.700 ± 0.020 ^a^
*L. Plantarum* 22699	0.970 ± 0.034 ^a^	1.508 ± 0.089 ^b^
*L. Plantarum* 23169	0.941 ± 0.034 ^ab^	1.633 ± 0.014 ^ab^

Note: All data are shown as the mean ± SD, and each value is a mean of five replicate determinations. Different letters in the same column indicate significant differences in the data (*p* < 0.05). The molecular order (DO) is ascertained by the peak area ratio of R1047/1022 cm^−1^, and the double helicity (DD) is expressed by the peak area ratio of R995/1022 cm^−1^.

**Table 3:** The amylose contents and chain length distributions of natural and fermented mung bean starch.

Sample	Amylose Content	Amylopectin Chain Length Distribution			
		DP (6–12) (FA)	DP (13–24) (Fb_1_)	DP (25–36) (Fb_2_)	DP (37–60) (Fb_3_)
Control	23.61 ± 0.21 ^c^	43.02 ± 0.86 ^b^	29.28 ± 1.08 ^a^	13.02 ± 0.08 ^a^	14.68 ± 0.14 ^a^
*L. Plantarum* YI-Y2013	35.36 ± 0.10 ^ab^	45.94 ± 0.35 ^ab^	27.98 ± 1.02 ^a^	12.92 ± 0.24 ^a^	13.16 ± 0.44 ^b^
*L. Plantarum* 22699	37.82 ± 0.33 ^a^	46.22 ± 1.77 ^a^	28.03 ± 1.11 ^a^	12.83 ± 0.26 ^a^	12.99 ± 0.31 ^b^
*L. Plantarum* 23169	33.01 ± 0.23 ^b^	46.66 ± 1.74 ^a^	28.31 ± 1.03 ^a^	12.68 ± 0.06 ^a^	13.37 ± 0.86 ^b^

All data are shown as the mean ± SD, and each value is a mean of three replicate determinations. Different letters in the same column indicate significant differences in the data (*p* < 0.05).

## Data Availability

The original contributions presented in the study are included in the article/Supplementary Material, further inquiries can be directed to the corresponding author.

## Supplementary Materials

The following supporting information can be downloaded at: https://www.mdpi.com/article/10.3390/foods13213409/s1, Table S1: The water absorption index (WAI), water solubility index (WSI), and swelling power (SP) of natural and fermented mung bean starch.
