# Supplementary material for: The Physicochemical Properties and Structure of Mung Bean Starch Fermented by Lactobacillus plantarum
Source: Foods. 2024 Oct 26;13(21):3409. doi: 10.3390/foods13213409 (PMC11545002; doi:10.3390/foods13213409)
Supplement: Supplementary file 1 [file foods-13-03409-s001.zip › foods-3240315-supplementary.pdf]

Table S1. The water absorption index (WAI), water solubility index (WSI), and swelling power (SP) of natural and fermented mung bean starch.

Index	Temperature/℃	Control	*L. plantarum* YI-Y2013	*L. plantarum* 22699	*L. plantarum* 23169
	55	4.06±0.3^{Ac}	2.93±0.2^{Cd}	3.62±0.21^{ABd}	3.25±0.27^{BCd}
	65	2.3±0.15^{Ad}	1.96±0.1^{Ae}	1.98±0.1^{Ae}	1.96±0.17^{Ae}
WAI	75	4.24±0.27^{Cc}	6.1±0.28^{Bc}	4.89±0.2^{Cc}	6.89±0.32^{Ac}
	85	7.81±0.36^{Bb}	8.52±0.3^{Ab}	8.77±0.26^{Ab}	8.95±0.3^{Ab}
	95	12.11±0.4^{Ba}	12.75±0.31^{Aa}	12.16±0.3^{Ba}	12.45±0.36^{ABa}
	55	1.05±0.1^{Ae}	0.71±0.06^{ABd}	0.75±0.05^{ABc}	0.64±0.06^{Bd}
	65	1.76±0.3^{Ad}	0.53±0.02^{BCd}	0.44±0.02^{Cc}	0.86±0.03^{Bd}
WSI	75	4.74±0.24^{Ac}	1.95±0.32^{Cc}	2.56±0.14^{Bb}	2.24±0.2^{BCc}
	85	5.87±0.3^{Ab}	3.38±0.15^{Bb}	2.8±0.2^{Cb}	2.6±0.2^{Cb}
	95	7.91±0.2^{Aa}	5.64±0.21^{Ba}	3.79±0.1^{Da}	4.81±0.1^{Ca}
	55	4.1±0.2^{Ad}	2.97±0.14^{Cd}	3.65±0.15^{Bd}	3.21±0.12^{Cd}
	65	2.31±0.1^{Ae}	1.99±0.1^{Ae}	1.99±0.1^{Ae}	1.98±0.12^{Ae}
SP	75	8.88±0.3^{Ac}	6.22±0.2^{Dc}	7.98±0.3^{Bc}	7.05±0.24^{Cc}
	85	9.8±0.32^{Ab}	7.79±0.25^{Cb}	8.71±0.2^{Bb}	8.04±0.2^{Cb}
	95	13.93±0.4^{Aa}	13.02±0.3^{Ba}	12.64±0.35^{Ba}	13.03±0.37^{Ba}

Note: In the table, different capital letters in the same row indicate significant differences in the corresponding indicators among different treatment groups at the same temperature; different lowercase letters in the same column indicate significant differences in the same treatment group with the increase in temperature ($p < 0.05$).
